# C-THAN: A new research center for the development of point-of-care technology for HIV/AIDS

**DOI:** 10.15641/ghi.v2i2.822

**Published:** 2019-11-27

**Authors:** Sally M. McFall, Mamoudou Maiga, Matthew R. Glucksberg, Kara M. Palamountain, Chad J. Achenbach, Robert L. Murphy

**Affiliations:** a Center for Innovation in Point-of-Care Technologies for HIV/AIDS at Northwestern University, Evanston, IL, USA; b Department of Biomedical Engineering, Northwestern University, Evanston, IL, USA; c University of Sciences, Techniques and Technologies of Bamako, Bamako, Mali; d Kellogg School of Management, Northwestern University, Evanston, IL, USA; e Department of Medicine, Northwestern University, Chicago, IL, USA

**Keywords:** HIV/AIDS, point-of-care, low-and-middle income countries, diagnosis

## Abstract

The Center for Innovation in Point-of-Care Technologies for HIV/AIDS at Northwestern University (C-THAN) is a partner in the Point-of-Care Technologies Research Network (POCTRN) of the National Institutes of Biomedical Imaging and Bioengineering. POCTRN’s mission is to drive the development of appropriate point-of-care (POC) diagnostic technologies through collaboration that merges scientific and technological capabilities with clinical need. C-THAN develops POC technologies for improved management of HIV/AIDS in low- and middle-income countries with a focus on sub-Saharan Africa. C-THAN incorporates clinical and user needs with technology expertise and resources to address commercialization and implementation barriers through: 1) assessment of unmet clinical needs in POC testing for HIV/AIDS and its comorbidities; 2) collaborations with physicians, researchers and engineers; 3) development of technical, clinical, industrial and regulatory partnerships; 4) clinical testing of prototype devices; and 5) creation of training opportunities for technology developers, evaluators, and other stakeholders. Technologies supported include tests for detection and monitoring of HIV/AIDS and its common comorbidities including tuberculosis, non-tuberculous mycobacteria, viral hepatitis and HIV-related malignancies. CTHAN relies on collaborations established by Northwestern University in Nigeria, South Africa, Mali and Tanzania, to have impact on the prevention and clinical management of HIV/AIDS.

## Introduction

The use of point-of-care (POC) diagnostics brings testing to or near the site of patient care, allowing immediate triage and treatment or discharge leading to improved clinical and/or economic outcomes (Pai et al., 2015). Accurate and timely diagnosis may also reduce empirical treatment based on patient symptoms which has contributed to the overuse of antibiotics and development of antibiotic resistance. POC testing can be performed in the home, at pharmacies and primary care clinics, in disaster and pandemic scenarios, in rural settings that lack laboratory services, in paramedical vehicles, and in hospital emergency departments, intensive care units and operating rooms (Chan et al., 2013). POC testing has played a crucial role in HIV diagnosis and treatment, especially in low- and middle-income countries (LMICs) in settings that lack access to formal laboratory services (Pai et al., 2015).

In 2006, the National Institute of Biomedical Imaging and Bioengineering (NIBIB) created the Point-of-Care Technologies Research Network (POCTRN) (https://cimit.org/ja/web/poctrn/home) to build multidisciplinary partnerships and expertise in the development of POC testing to address unmet clinical needs (Ford Carleton et al., 2016). For more than a decade, NIBIB has supported specialized centers through cooperative agreements (the U54 funding mechanism) to identify clinical needs, as well as guide and support technology development and appropriate clinical testing. Recently, a third solicitation funded five centers, one of which is The Center for Innovation in Point-of-Care Technologies for HIV/AIDS at Northwestern University (C-THAN). C-THAN is funded by NIBIB, the Fogarty International Center, and the Office of AIDS Research to develop POC technologies critical for improved management of individuals with HIV/AIDS and to facilitate technology commercialization in LMICs with specific emphasis on sub-Saharan Africa.

## C-THAN and clinical needs

In 2016, HIV/AIDS accounted for 1.03 million deaths globally, while 1.21 million deaths were attributable to tuberculosis (TB), making these the leading causes of mortality due to infectious diseases worldwide (UNAIDS, 2017). The majority of persons with HIV and its co-morbidities (such as TB) reside in LMICs where timely diagnosis and prompt treatment of these conditions are constrained by limited laboratory support (Lahuerta et al., 2013). Despite the bleak nature of these figures, they actually represent significant improvements in mortality rates from just over 10 years ago. In 2005, 1.9 million deaths were attributable to HIV/AIDS (WHO, 2016a) and more than 1.5 million deaths to TB (WHO, 2017). The 45% decrease in HIV mortality observed was the result of improvements in diagnostic tools and a massive increase in access to antiretroviral therapy (ART). Likewise, there has been > 50% decrease in the number of new infections each year from 2000 to 2010 most likely caused by reduction in risky behavior (WHO, 2016b). Inspired by this success, the United Nations’ Program on HIV and AIDS (UNAIDS) set the ambitious 90-90-90 goals to achieve detection of 90% of HIV cases, treatment for 90% of those cases, and viral suppression for 90% of those treated, by 2020 (UNAIDS, 2017). These goals will not be achieved in LMICs without the addition of POC diagnostic tests that are affordable, rapid, easy to use, and require little maintenance (Arora et al., 2013).

**Figure 1.**
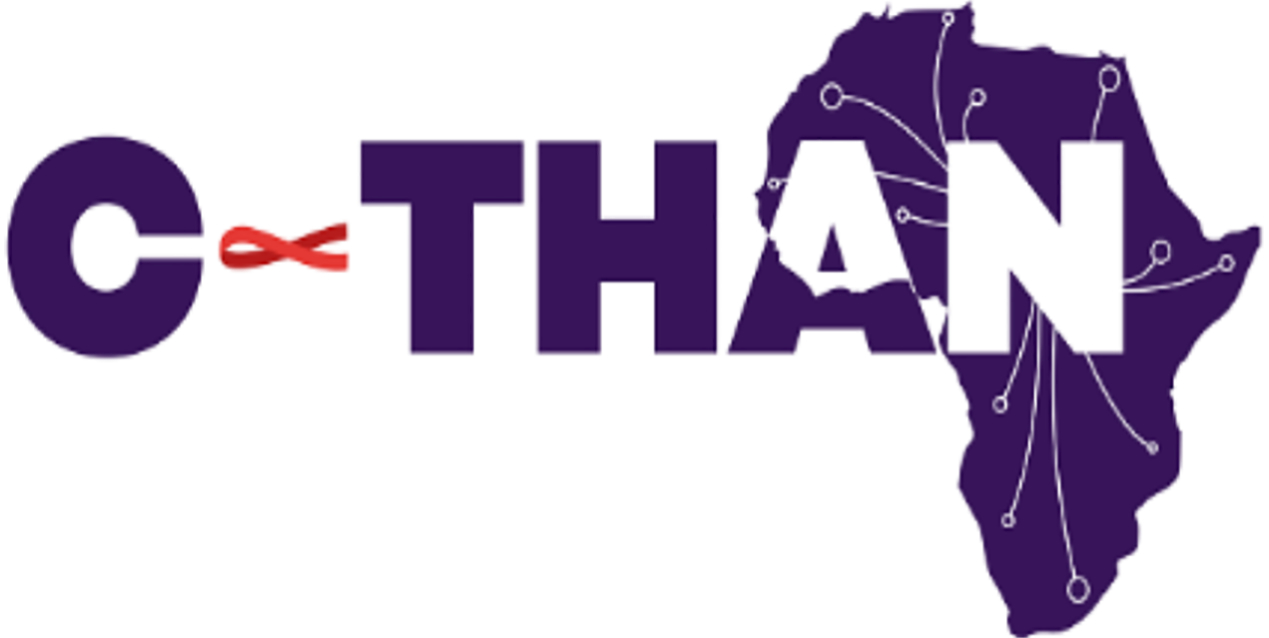
Logo of C-THAN. Designed to illustrate C-THAN’s commitment to serving patients living with HIV in Africa.

C-THAN will support the development of such technologies, facilitate their translation into high quality clinical applications, and importantly promote their production and commercialization in LMICs. To create C-THAN, we have assembled a scientific consortium of clinical and biomedical engineering expertise with a 17-year history of collaboration in addressing infectious diseases in sub-Saharan Africa which includes Northwestern University, Muhumbili University, Stellenbosch University, University of Bamako, University of Cape Town, University of Ibadan, University of Jos, and University of Lagos. We have designed a logo for C-THAN (Figure 1) to represent our extensive research network and to aid with public recognition.

C-THAN’s resources are designed to bridge the gap between biomedical engineering and global health, supporting technological innovation for improved health and healthcare, with an emphasis on developing settings. C-THAN is dedicated to building partnerships between United States and LMIC investigators and prioritizes collaborations with LMIC technology developers.

## C-THAN’s areas of interest

POC rapid diagnostic tests (RDTs) primarily using lateral flow assays to detect HIV antibodies have been in use in LMICs for nearly 20 years, with 27 commercial *in vitro* diagnostic tests having received World Health Organization prequalification (WHO, 2018). They have facilitated diagnostic testing outside laboratory settings and thus contributed to the reduction in mortality from HIV/AIDS (Pai et al., 2015; Reid et al., 2013); this has supported the scale-up of ART to decentralized clinics. Health care can be transformed in LMICs by life-saving health services reaching communities that were previously inaccessible. However, technological advances and early opportunities to test in “real-world” settings during development are still needed to improve the quality of HIV diagnostics.

Testing POC technologies (POCTs) in research settings has overestimated the accuracy of diagnostic tests and does not necessarily consider environmental conditions specific to low resource settings (Drain et al., 2014). For a test to be successful in the lab with trained personnel, a good sensitive/specific assay is commonly what is required. In the field, several mistakes are common in the use of RDTs (Villadiego, 2012) including improper timing, adding too much buffer, inaccurate reading of test lines and poor-quality assurance practices. Technologies that overcome these errors include high contrast test lines to enhance readability; integrated timers to assure proper incubation time; unit doses of buffer; and integrated cellular network access to allow communication of results for quality assurance and logistics.

In addition to HIV serology, other tests that are essential for treatment initiation and monitoring and drug sensitivity testing are not yet readily available at the POC. These tests are critical for identifying infections earlier, preventing onward transmissions, and thus reducing HIV incidence at a population level. To increase people’s awareness of their HIV status, non-symptomatic persons especially those in at risk populations (e.g., men having sex with men, sex workers and people who inject drugs) should be screened frequently with tests that detect HIV infection before antibodies can be detected. Upon diagnosis, ART is to be initiated immediately (WHO, 2015), and viral load quantification should commence as it is the most reliable biological indicator for treatment monitoring (Usdin et al., 2010). In addition, with increased access to ART, the need for additional diagnostic tools for POC HIV drug resistant testing has arisen (Duarte et al., 2017).

C-THAN will incorporate clinical and user needs in the development process for POC tests while providing access to expertise and resources to address early challenges to commercialization and implementation. The range of POC technology applications will include detection and monitoring of HIV/AIDS infection and its common fatal comorbidities including tuberculosis, infections of non-tuberculous mycobacteria, hepatitis B, hepatitis C and HIV-related malignancies.

## C-THAN’s organization

**Figure 2.**
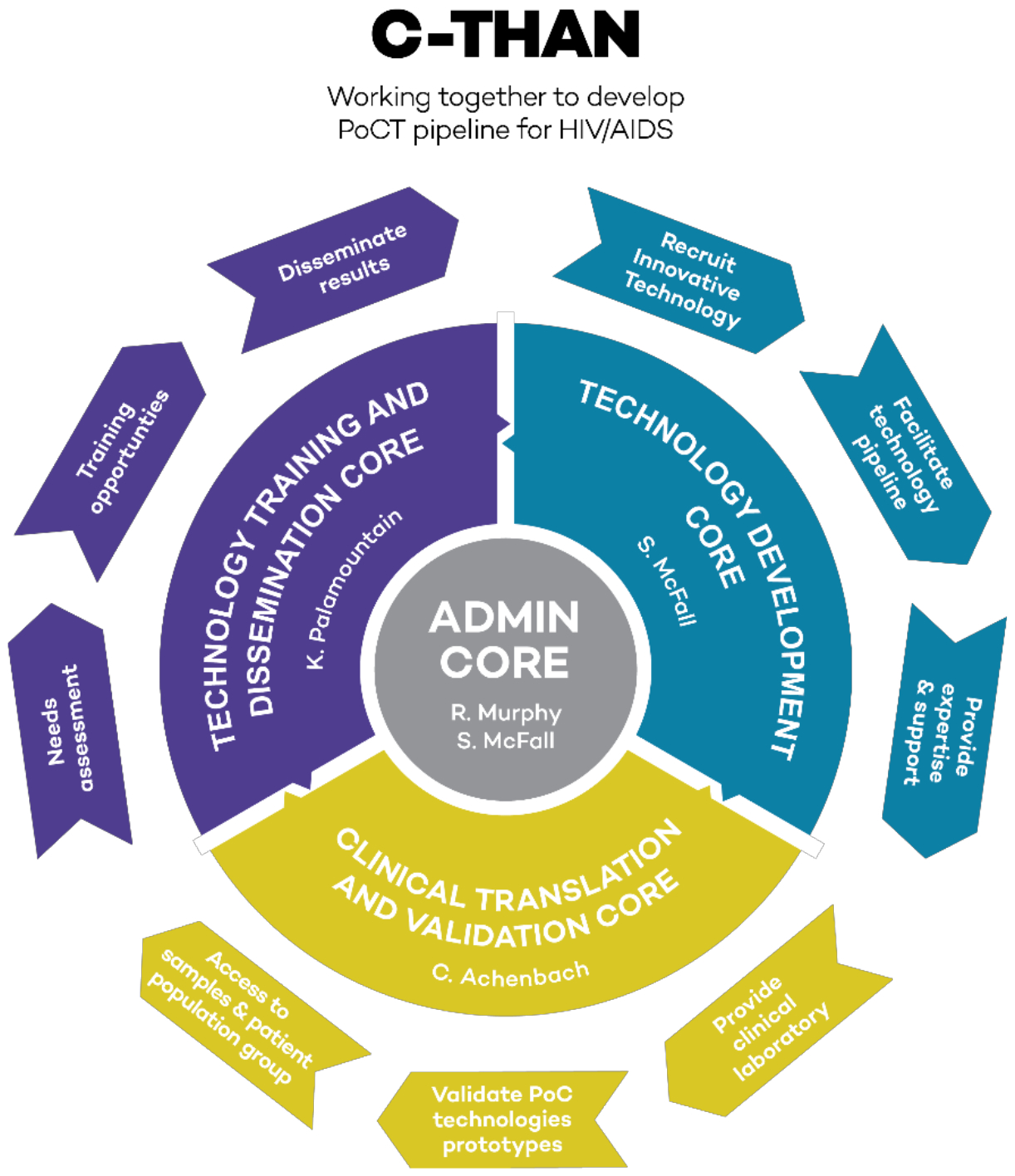
Four interconnected C-THAN cores.

C-THAN’s efforts entail: 1) assessment of unmet clinical needs in POC testing for HIV/AIDS and its comorbidities; 2) collaboration with relevant scientists, physicians, researchers and engineers; 3) development of essential technical, clinical, industrial and regulatory partnerships; 4) testing of prototype POC devices in the field; and 5) creation of training opportunities for technology developers, evaluators, and other stakeholders. Product development requires the coordinated activity of the four C-THAN cores: Administrative, Technology Training and Dissemination, Technology Development/Refinement, and Clinical Translation and Validation as illustrated in Figure 2.

**Figure 3.**
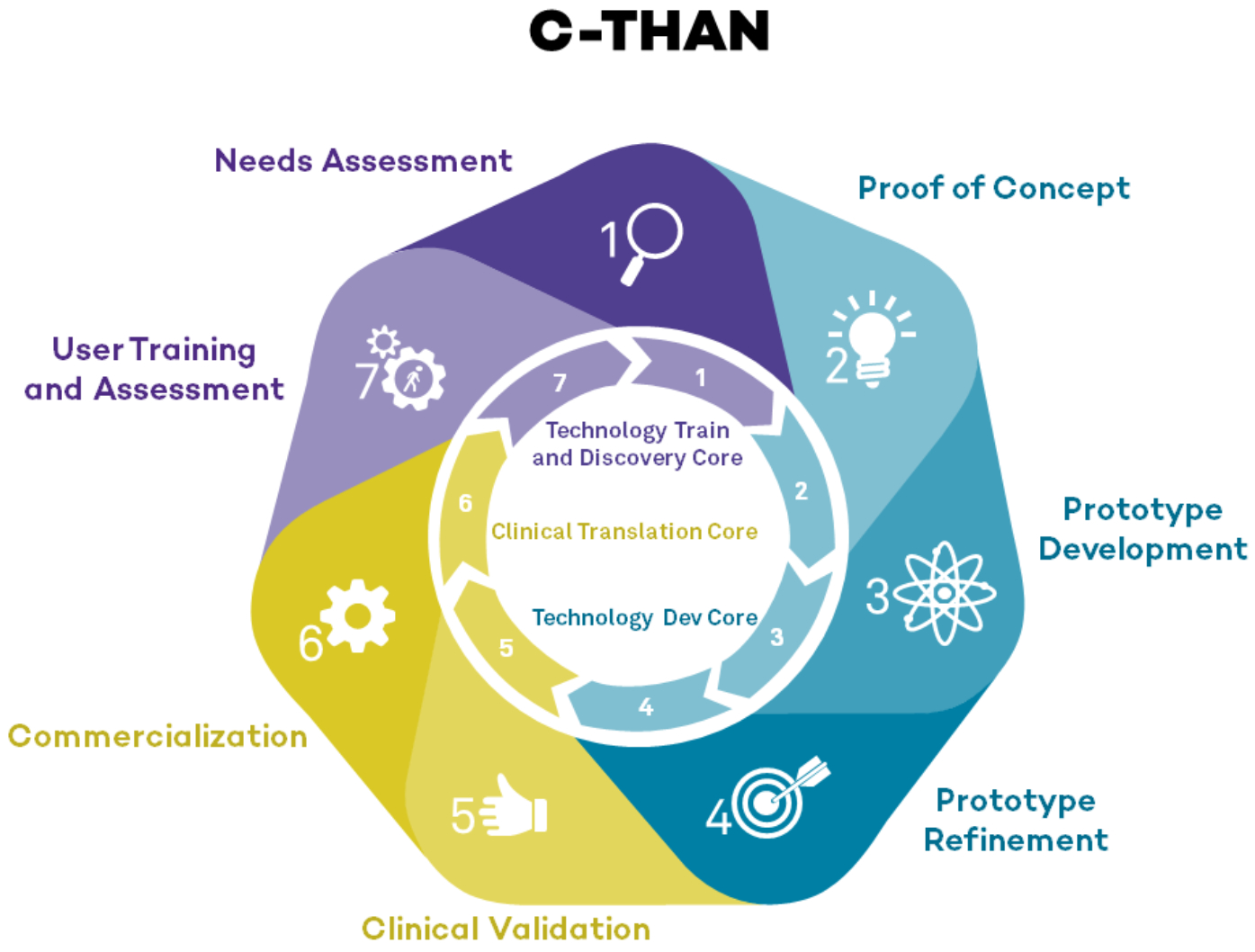
Product development support through the concerted effort of C-THAN cores.

*Administrative Core* maintains the operational and strategic structure to manage C-THAN as a distinct cohesive entity that integrates, supports and coordinates partner and Core activities and supported projects. The Administrative Core leads the organization and governance of C-THAN, all budgetary matters, collaboration with POCTRN, communication with all stakeholders, and program/project monitoring and evaluation. Product development and commercialization requires the coordinated efforts of the Technology Development, Clinical Translation and Validation, and Technology Training and Dissemination Cores (Figure 3).

*Technology Development Core* identifies, evaluates and supports POCT development projects both internal and external to C-THAN. Specifically, we solicit and select meritorious POCT projects promoting high priority HIV/AIDS topics and implement a technology development/refinement and testing plan for supported projects.

*Clinical Translation and Validation Core* provides a “clinical laboratory” for innovators that focuses on validation, adoption, feasibility and implementation of POCT. Depending on the maturity of the technology, we review projects in the Technology Development Core for potential transition into clinical validation projects. Our primary goal is to provide the clinical infrastructure and services necessary to ensure that the POCT prototypes supported under this program will have a high rate of success.

*Technology Training and Dissemination Core* draws from resources at the Northwestern University Kellogg School of Management in teaching and conducting needs assessments for POCT. Through this Core, researchers and developers of POCT are coached in all aspects of innovation, including needs assessment, clinical evaluation, critical business aspects such as manufacturing, regulatory requirements, and dissemination of their products. We facilitate interdisciplinary training on the development, evaluation, implementation, and commercialization of POCT related to HIV/AIDS in LMICs; conduct needs assessments of POCT for HIV/AIDS infected patients in LMICs; and define the geographical and clinical scope for needs assessments.

C-THAN has seven clinical sites in sub-Saharan Africa: Universities of Lagos, Ibadan and Jos (Nigeria), University of Cape Town and Stellenbosch University (South Africa), University of Sciences, Techniques and Technologies Bamako (Mali) and Muhimbili University of Health and Allied Sciences (Tanzania). C-THAN also has three biomedical engineering sites: Universities of Lagos, Ibadan and Cape Town. Investigators at these sites collaborate with Northwestern University faculty to provide the in C-THAN core functions described above and are available for individual collaborations. C-THAN provides opportunities for interactions with core members including potential collaborations in clinical needs assessment, conducting clinical validation studies, technology implementation, network building, and mentorship in project development, technology development, designing for future manufacturing, and grant writing.

## C-THAN’s solicitation plan for pilot projects

In each of the five years of the project, one-year technology development grants of approximately $100,000 will be awarded for HIV/AIDS related assay/device development projects. The grants must address the NIH’s high priority topics for HIV/AIDS research, including: reducing HIV incidence by improving screening, detection, treatment monitoring, and HIV drug resistance detection; diagnosing HIV-associated comorbidities including tuberculosis, non-tuberculosis mycobacteria, hepatitis B, hepatitis C and HIV-associated cancers; reducing health disparities by developing testing technology for underserved community settings; and training of the workforce to translate POCT from research & development to implementation.

Six projects were selected for funding in 2019 (year two). Combined with the seven pilot projects in the original grant application, C-THAN’s research portfolio currently covers *in vitro* diagnostic assays and technologies, treatment related diagnostic technologies, and technologies that improve or enable POC test performance. Essential product attributes include: meeting clinical needs of people living with HIV/AIDS; being operable in settings with limited medical infrastructure; and being simple to operate, durable, and manufacturable at low cost and with low-cost disposables.

Development grant awards for years three to five will go through a two-stage process. First, there will be a two-page pre-proposal (expression of interest) submitted with a description of the unmet clinical need, proposed innovation and collaborators, which will be triaged by C-THAN leadership with oversight of NIH scientific officers. The second phase will be submission of invited full proposals. The solicitation for proposals will be published on the POCTRN website (https://cimit.org/ja/web/poctrn/home).

The content of the proposals will include a discussion of the unmet need and a description of the proposed solution including advantages over the alternatives and the maturity of the proposed solution. The work plan will include a table of deliverables and the implementation pathway. The application will also include a description of the team and resources, budget and justification. Funded projects that achieve their proposed milestones will be encouraged to apply for follow-on funding and/or clinical evaluation studies.

## Conclusion

Meeting UNAID’s HIV 90-90-90 goals requires new approaches to successfully transform healthcare delivery to people living with HIV/AIDS. C-THAN’s novel partnership model supports collaborations across disciplines, institutions and geographic regions and aims to drive innovative POC solutions. C-THAN seeks collaborators for technology development, clinical implementation, manufacturing and regulatory processes.
